# *Agrimonia procera* Wallr. Extract Increases Stress Resistance and Prolongs Life Span in *Caenorhabditis elegans* via Transcription Factor DAF-16 (FoxO Orthologue)

**DOI:** 10.3390/antiox7120192

**Published:** 2018-12-14

**Authors:** Christina Saier, Inge Gommlich, Volker Hiemann, Sabrina Baier, Karoline Koch, Gert Horn, Tanja Kowalewsky, Jörg Bartelt, Maria Seemann, Wim Wätjen

**Affiliations:** 1 Biofunctionality of Secondary Plant Compounds, Institute of Agricultural and Nutritional Sciences, Martin-Luther-University Halle-Wittenberg, Weinbergweg 22, 06120 Halle (Saale), Germany; christina.saier@landw.uni-halle.de (C.S.); ingegommlich@web.de (I.G.); volker.hiemann@student.uni-halle.de (V.H.); sabrina.baier@landw.uni-halle.de (S.B.); karoline.koch@landw.uni-halle.de (K.K.); 2 Exsemine GmbH, Am Wehr 4, 06198 Salzatal, Germany; g.horn@exsemine.de; 3 Kaesler Nutrition GmbH, Zeppelinstraße 3, 27472 Cuxhaven, Germany; Tanja.Kowalewsky@kaesler.de (T.K.); Joerg.Bartelt@kaesler.de (J.B.); Maria.Seemann@kaesler.de (M.S.)

**Keywords:** aging, antioxidant, life span-extending effects, insulin-like signaling, oxidative stress, phytochemicals

## Abstract

*Agrimonia procera* is a pharmacologically interesting plant which is proposed to protect against various diseases due to its high amount of phytochemicals, e.g., polyphenols. However, in spite of the amount of postulated health benefits, studies concerning the mechanistic effects of *Agrimonia procera* are limited. Using the nematode *Caenorhabditis elegans*, we were able to show that an ethanol extract of *Agrimonia procera* herba (eAE) mediates strong antioxidative effects in the nematode: Beside a strong radical-scavenging activity, eAE reduces accumulation of reactive oxygen species (ROS) accumulation and protects against paraquat-induced oxidative stress. The extract does not protect against amyloid-β-mediated toxicity, but efficiently increases the life span (up to 12.7%), as well as the resistance to thermal stress (prolongation of survival up to 22%), of this model organism. Using nematodes deficient in the forkhead box O (FoxO)-orthologue DAF-16, we were able to demonstrate that beneficial effects of eAE on stress resistance and life span were mediated via this transcription factor. We showed antioxidative, stress-reducing, and life-prolonging effects of eAE *in vivo* and were able to demonstrate a molecular mechanism of this extract. These results may be important for identifying further molecular targets of eAE in humans.

## 1. Introduction

The agrimony herb is a traditional plant drug, which is commonly used as an astringent agent, as well as medication for inflammatory, cardiovascular, and other oxidative-related diseases [1]. The genus *Agrimonia* L. (*Rosaceae, Rosoideae*) comprises 16 species of perennial herbs distributed in the northern hemisphere [1]. *Agrimonia procera* Wallr., commonly known as fragrant agrimony, is a well-distinguished taxon growing in similar habitats to *A. eupatoria* and is usually considered to be less common [2]. According to European Pharmacopoeia, the only source of this plant drug is *Agrimonia eupatoria*. By contrast, the German Commission E pharmacopoeial monograph is employed to allow *Agrimonia procera* to be used as a second valid source of *Agrimoniae* herba. Different effects were shown for *Agrimonia* spp.: for example, *Agrimonia pilosa* Ledeb. possesses several physiological activities, such as anti-cancer, antioxidant, anti-inflammatory, hepatoprotective, anti-Alzheimer, and anti-diabetic properties. The data on the phytochemistry of *A. procera* are scarce [1]. Pukalskienė et al. emphasized that the most important medicinal application of *A. procera* might be protection of the cardiovascular system against menopause-induced changes [3]. Gräber et al. showed that low dosages of *Agrimonia procera* provide beneficial effects on feed intake in piglets [4] and exert antimicrobial effects, as well as increase the immune response, in lipopolysaccharide-challenged piglets [5].

Several studies have been conducted on the phytochemical composition of *Agrimonia* spp.: Polyphenols are major constituents, including phenolic acids, flavonoids, ellagitannins, and procyanidins. These compounds are strong radical scavengers and may be responsible for the comparatively high antioxidant potential of this plant. Agrimoniin is reported as a major ellagitannin occurring in common agrimony in high quantities. Phytochemical investigations of *A. procera* are scarce, but it was shown that this agrimony species contains agrimoniin, and flavonoids generally defined as luteolin and apigenin glycosides [1,3]. It is suggested that these proposed beneficial health effects are attributable to these phytochemicals [1,3].

A wide spectrum of pharmacological effects of *Agrimonia* spp. has been reported, but mechanistic *in vivo* reports about the antioxidative, antidiabetic, and anti-Alzheimer effects of the extract, as well as studies showing protection against stress, are limited [1]. Therefore, we analyzed the effects of an ethanolic *Agrimonia procera* extract (eAE) in the model organism *C. elegans*. In our study, we focused on antioxidant effects *in vivo*, stress resistance, protection against amyloid-β, and life span modulating effects. These results are important for the identification of molecular targets of eAE in humans (pharmacological use).

## 2. Materials and Methods

**Figure 1:**
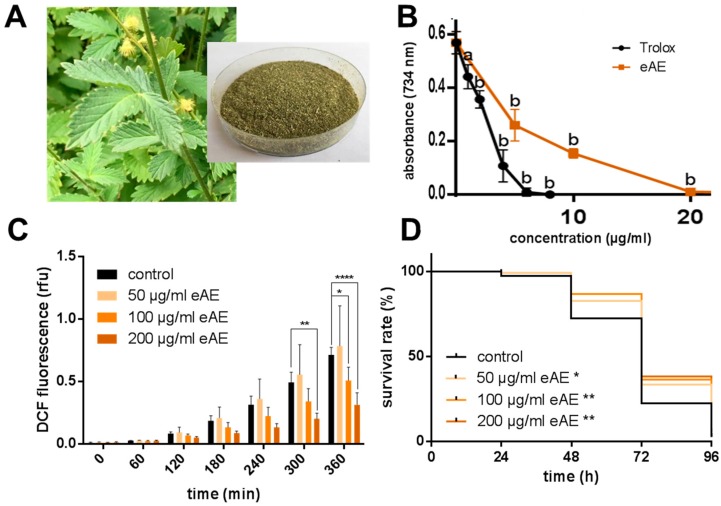
Radical-scavenging and antioxidative effects of eAE. (**A**) Image of *Agrimonia procera* plant/dried plant material; (**B**) Radical scavenging activity (TEAC assay): In this cell-free assay, eAE shows antioxidant properties. Values are mean ± SD, n = 3, one-way ANOVA with Dunnett’s multiple comparisons test vs. control (0 µg/mL), a: ** *p* ≤ 0.01, b: **** *p* ≤ 0.0001; (**C**) Thermally induced increase in ROS (DCF assay): Wild type L4 larvae were treated with different concentrations of eAE or with DMSO (vehicle, 0.4%) for 24 h (20 °C), and were then transferred individually into the wells of a 384-well plate containing 50 µM H_2_DCF-DA. The increase in fluorescence was measured at 37 °C. Values are mean ± SD, n = 3 (each 16 individuals per group), two-way ANOVA with Dunnett’s multiple comparisons test vs. control at the correspondent time point, * *p* ≤ 0.05, ** *p* ≤ 0.01, **** *p* ≤ 0.0001; (**D**) Oxidative stress resistance: Wild type L4 larvae were incubated with eAE or with DMSO (vehicle, 0.4%) for 72 h at 20 °C, and all nematodes were then transferred into extract-free media containing 50 mM paraquat. The survival of the nematodes was surveyed via touch-provoked movement every 24 h. Kaplan-Meier statistics were used for the comparison of the survival curves, n = 3 (each 40 individuals per group), Log Rank (Mantel-Cox) test, * *p* ≤ 0.05, ** *p* ≤ 0.01. Calculated data for oxidative stress resistance is shown in Supplementary Table S3.

Materials: Trolox was purchased from Calbiochem (Merck, Darmstadt, Germany) and SYTOX^®^ Green Nucleic Acid Stain was obtained from Molecular Probes Inc. (Leiden, The Netherlands). All other chemicals were of analytical grade and were purchased from SIGMA-Aldrich (Taufkirchen, Germany). *Agrimonia procera* “Magna” (harvested: June 2017) was provided by Exsemine GmbH (Salzatal, Germany), and the eAE was obtained by extraction of the dried plant material (*Agrimonia procera herba*, polyphenol content: 5.54%, agrimoniin: 1.91%, Figure 1A) using 100% ethanol (reflux, 1 h). The ethanolic extract was vaporized and the residue was completely dissolved in DMSO (stock solution: 250 mg/mL). In each experiment, the same amount of the vehicle (DMSO, 0.4%) was used.

*C. elegans* strains and maintenance: *C. elegans* strains used in this study (wild-type N2 var. Bristol, CF1038 [*daf-16*(*mu86*) I.], TJ356 [zIs356 IV (*daf-16p::daf-16a/b*::GFP+*rol-6*)], CL4176 [*smg-1*(*cc546*) I; dvls27 X.]) and the bacterial strains were provided by the *Caenorhabditis* Genetics Center (CGC). Strain maintenance was performed at 20 °C on nematode growth medium (NGM) agar plates containing a lawn of *E. coli* var. OP50 as the food source. Compound treatment of *C. elegans* was conducted in 1.5 mL of liquid NGM containing 1% (w/v) bovine serum albumin, 12.5 µg/mL tetracycline, 100 µg/mL ampicillin, and *E. coli* OP50-1 (10^9^ cfu/mL) in 35 mm petri dishes. Stock solution (250 mg/mL) of eAE was prepared in DMSO and concentrations of 50, 100, and 200 µg/mL eAE were used for each experiment. Age synchronous nematodes were obtained by treating gravid adults with a bleaching solution (50% NaOH (5 M)/50% NaClO (13%)), followed by three washing steps in liquid NGM. The remaining eggs were allowed to hatch on fresh NGM agar plates seeded with OP50. After three days at 20 °C, an age synchronous population of L4 larvae and young adult animals were used for experiments. For the paraquat assay, an age synchronous population was generated by timed egg-laying: Gravid adult nematodes were transferred on NGM agar plates containing a lawn of *E. coli* var. OP50 and were allowed to lay eggs. After 2 h, all adult animals were removed from the plate and the eggs were incubated for three days at 20 °C. For the Aβ assay, synchronization was performed by egg laying in liquid NGM media with *E. coli* OP50-1 (10^9^ cfu/mL).

Antioxidative capacity *in vitro* (TEAC assay): The trolox equivalent antioxidative capacity (TEAC) assay is a cell-free method for the detection of radical-scavenging properties of test compounds in comparison to the synthetic vitamin E derivative trolox (positive control). The ABTS stock solution (2,2′-azino-bis(3-ethylbenzothiazoline-6-sulphonic acid), a stable colored radical) was stored in the dark overnight and subsequently diluted with ethanol to obtain an absorption of 0.6 at 734 nm. Aliquots (10 µL) of eAE or trolox in various concentrations were pipetted into the wells of a transparent 96-well microtiter plate, mixed with 290 µL of the radical solution, and stored at room temperature for 12 min to start the reaction. The absorption was measured spectrophotometrically at 734 nm.

Measurement of intracellular ROS accumulation *in vivo*: Synchronized L4 larvae and young adults (N2 and CF1038) were treated with different eAE concentrations for 24 h, washed in phosphate buffered saline/0.1% Tween 20 (PBST), and transferred individually in 1 µL PBST into the wells of a 384-well microtiter plate. The wells contained 7 µL M9 buffer. After the transfer of all nematodes, 2 μL of 250 μM H_2_DCF-DA was added to each well and the plate was sealed to prevent evaporation. During thermal stress (37 °C), fluorescence intensities (excitation: 485 nm; emission: 535 nm) were recorded with a fluorescence reader. Fluorescence values were normalized to the increase of the control value (t = 7 h).

Oxidative stress resistance (Paraquat assay): Synchronized L4 larvae and young adults were treated for three days, with different concentrations of eAE or with DMSO as a control and with FUDR (5-fluorodeoxyuridine) to prevent progeny from hatching. Then, the nematodes were transferred into eAE-free medium containing 50 mM paraquat. For the following four days, the survival of the nematodes was analyzed by touch-provoked movement every 24 h. This experiment was carried out with wild-type nematodes and with the loss-of-function mutant strain CF1038.

Thermal stress resistance (SYTOX^®^ Green assay): Synchronized L4 larvae and young adult animals (N2 and CF1038) were treated with eAE (see above) for 24 h. Prior to the application of thermal stress, animals were washed in PBST. After transferring each nematode in 1 µL of PBST into a well of a 384-well microtiter plate containing 9 µL PBS, SYTOX^®^ Green Nucleic Acid Stain in PBS (10 μL of a 2 μM solution) was added and the wells were sealed to avoid evaporation. Thermal stress (37 °C) was applied and the increase of fluorescence which correlates with an increase in cells with disturbed membrane integrity was measured (excitation: 485 nm; emission: 535 nm) with a fluorescence reader. When the fluorescence values of individual nematodes exceeded a defined cut-off value (mean fluorescence values of the first four measurements multiplied by the factor three), the corresponding animal was scored as dead. The description of the death criteria was adopted from Gill et al. (2003) [6] and verified by manual analysis (touch-provoked movement).

Life span: For the analysis of the life span at 25 °C, the wild-type strain N2, as well as CF1038, was used. Forty synchronized L4 larvae and young adult animals were transferred into liquid media (as described above). This time point was considered as day 0 of the life span. During the first 10 days of the life span, the media contained 120 µM FUDR to prevent the hatching of viable progeny. The incubation media were exchanged every day and the survival of the animals was measured by touch-provoked movement. 

Aβ Assay: Eggs of the transgenic strain CL4176 were incubated with different concentrations of eAE or DMSO as a control or caffeine (5 mM, [7]) as a positive control for two days. L3 larvae were transferred from the media onto agar plates containing a lawn of *E. coli* var. OP50 and stored at 25 °C. After 26, 28, 30, 32, and 34 h, all nematodes were tested by touch-provoked movement. Nematodes only slightly moving their head or not moving at all were scored as paralyzed.

Intracellular localization of DAF-16::GFP: The transgenic strain TJ356 was used to detect the intracellular localization of the GFP-tagged transcription factor DAF-16. Synchronized L4 larvae and young adult animals of the corresponding strains were transferred into liquid treatment media (as described above) and maintained for one hour at 20 °C, respectively. Subsequently, a drop of 9 µL medium containing the nematodes was placed on a microscope slide and mixed with 9 µL of the anesthetic levamisole (20 mM). After the application of a cover slip, the cellular localization of DAF-16::GFP in the whole body was detected by fluorescence microscopy. 

Statistics: Statistical analyses were conducted for at least three independent experiments, with data given as mean ± SD. PASW Statistics for Windows, Version 18.0 (SPSS Inc.; Chicago, IL, USA) and GraphPad Prism 6 (La Jolla, CA, USA) software was used to compute the statistical analyses. Statistical significance was determined by one-way or two-way Analysis of variance (ANOVA) with Dunnett’s or Tukey’s post hoc-test, while life span, oxidative, and thermal stress resistance, as well as Aβ paralysis curves, were calculated using Kaplan-Meier survival analyses with a log-rank test. Statistical differences were considered to be significant at a level of *p* ≤ 0.05.

## 3. Results

### 3.1. eAE Scavenges Radicals and Protects against Oxidative Stress

We analyzed the radical-scavenging effects of eAE in comparison with the well-known radical-scavenging compound trolox, a synthetic vitamin E derivative [8]. The ethanolic extract showed a strong antioxidative capacity *in vitro* (Figure 1B). The IC_50_-values of the radical-scavenging activity were determined as 4 µg/mL (trolox: 2 µg/mL). To investigate if these radical-scavenging effects are relevant *in vivo*, we used the model organism *Caenorhabditis elegans*. First, we induced the formation of ROS in the nematode by the application of thermal stress (37 °C). Using DCF, a fluorescent probe for ROS, a strong increase in ROS formation inside the nematodes was detected during thermal stress (Figure 1C): Basal DCF fluorescence (0 min: 0.0086 rfu) increased about 80-fold (360 min: 0.7136 rfu). Pre-incubation with eAE (50, 100, and 200 µg/mL) showed that only the two highest concentrations, and not 50 µg/mL, were able to decrease ROS-formation by 28.8 and 55.9%, respectively (360 min). In a second approach, ROS were generated by the application of paraquat, a compound which acts as a redox-cycler continuously generating superoxide anions. The amount of living nematodes was monitored every 24 h by touch-provoked movement. In this experiment, all three concentrations analyzed (50, 100, and 200 µg/mL) caused a significant increase in survival against this kind of oxidative stress (Figure 1D).

### 3.2. eAE Increases Life Span and Thermal Stress Resistance but Causes no Resistance against Amyloid-β Toxicity

**Figure 2:**
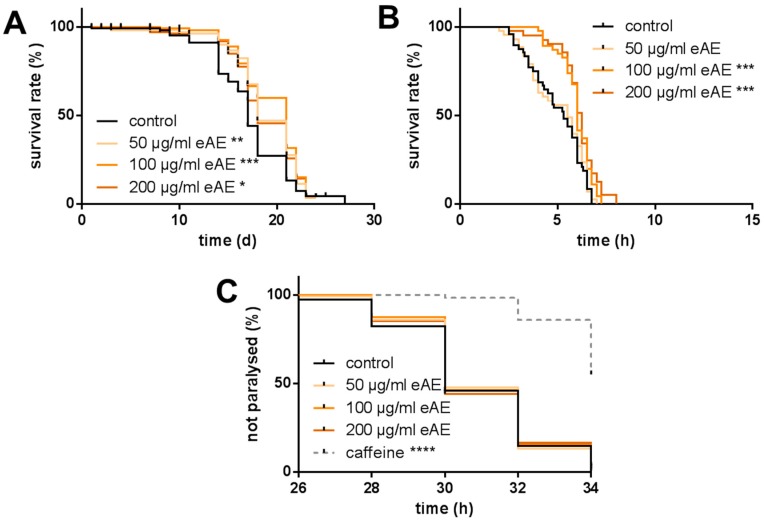
Effects of eAE on life span and resistance against thermal and amyloid-β-stress. (**A**) Modulation of life span: Wild type L4 larvae were incubated with different concentrations of eAE or DMSO (vehicle, 0.4%) at 25 °C. On five days a week, medium was exchanged and survival of the nematodes was tested via touch-provoked movement. Kaplan-Meier statistics were used for the comparison of the survival curves, n = 3 (each 40 individuals per group), Log Rank (Mantel-Cox) test, * *p* ≤ 0.05, ** *p* ≤ 0.01, *** *p* ≤ 0.001; (**B**) Thermal stress resistance: Wild type L4 larvae were treated with different concentrations of eAE or with DMSO (vehicle, 0.4%) for 24 h at 20 °C, and then transferred individually into the wells of a 384-well plate containing 1 µM SYTOX^®^ Green. The increase in fluorescence was measured at 37 °C. The graph shows the percentage of viable nematodes. Kaplan-Meier statistics were used for the comparison of the survival curves, n = 3 (each 16 individuals per group), Log Rank (Mantel-Cox) test, *** *p* ≤ 0.001; (**C**) Toxicity of Aβ: Transgenic nematodes of the strain CL4176 were incubated as eggs with different concentrations of eAE, DMSO (vehicle, 0.4%) or caffeine as a positive control at 16 °C. L3 larvae were transferred on agar plates containing a lawn of *E. coli* var. OP50 and kept at 25 °C. After 26, 28, 30, 32, and 34 h, nematodes were analyzed for occurrence of paralysis. Kaplan-Meier statistics were used for the comparison of the survival curves, n = 3 (each 40 individuals per group), Log Rank (Mantel-Cox) test, **** *p* ≤ 0.0001. Calculated data for the experiments are shown in Supplementary Tables S1, S2, and S4.

Since the antioxidative effects of phytochemicals and plant extracts often result in a prolongation of life span, we analyzed the effects of eAE on longevity. All three concentrations (50, 100, and 200 µg/mL) significantly increased the mean life span (prolongation compared to the control nematodes of 8.1, 12.7, and 7.5%, respectively, Figure 2A). Since we demonstrated that the application of eAE was able to reduce the thermally induced ROS generation in the nematode (Figure 1C), we then investigated if the treatment with the extract also results in protection of the nematodes against lethal thermal stress. The death of the nematodes was detected via SYTOX^®^ Green fluorescence and for each single nematode, the virtual death point was calculated and plotted in a graph. The mean life span of the thermally stressed nematodes was determined to be 5.0 ± 0.196 h (Figure 2B). Consistent with the results obtained in the DCF assay (Figure 1B), only 100 and 200 µg/mL, and not 50 µg/mL, eAE resulted in significant protection against thermal-induced lethal stress. Next, we investigated if eAE also protects the nematode against amyloid-β toxicity: Transgenic nematodes expressing the human amyloid-β peptide start to become paralyzed approximately 26 h after temperature-induced transgene induction. However, pre-incubation with different concentrations of eAE (50, 100, and 200 µg/mL) caused no protection from toxic amyloid-β-stress. The positive control used for this assay (caffeine, 5 mM) caused a significant delay in the paralysis time: more than 50% of the animals were freely moving, even after 34 h of amyloid-β-stress (Figure 2C).

### 3.3. Modulation of DAF-16 Localization by eAE

**Figure 3:**
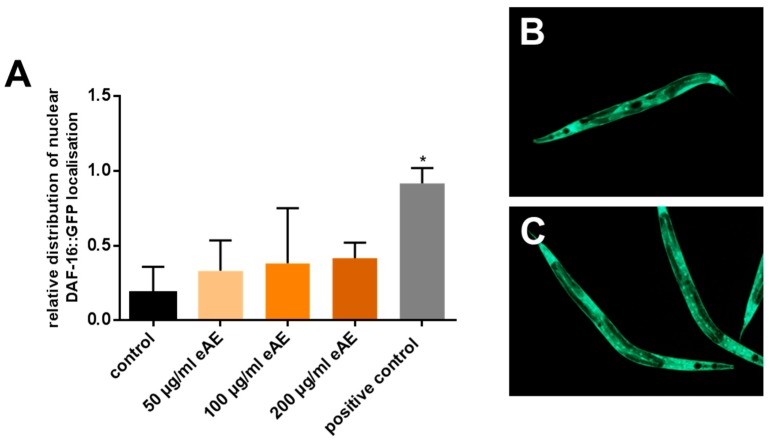
Effects of eAE on the intracellular localization of DAF-16. (**A**) Intracellular localization of DAF-16: L4 larvae of the transgenic strain TJ356 (DAF-16::GFP) were incubated with different concentrations of eAE or DMSO (vehicle, 0.4%) for 1 h at 20 °C or kept for five minutes at 37 °C (positive control), and localization of the transcription factor was then examined using fluorescence microscopy. Values are mean ± SD, n = 3 (each 20 individuals per group, one-way ANOVA with Tuckey’s multiple comparisons test vs. control, * *p* ≤ 0.05; (**B**) representative image for a nematode with mainly cytosolic localization of DAF-16; (**C**) representative image for nematodes with mainly nuclear localization of DAF-16.

Next, we investigated molecular mechanisms that are modulated by eAE to cause the protective effects in *C. elegans*. Therefore, we focused on the pivotal transcription factor DAF-16 (mammalian FoxO orthologue). Using *C. elegans* strains expressing DAF-16::GFP fusion proteins, we were able to analyze the cellular localization of this factor: When the GFP-fluorescence of an individual nematode is mainly diffuse, this animal is categorized as a nematode with “cytosolic” (=inactive) localization of this transcription factor (Figure 3B). Additionally, distinct GFP fluorescence (dots, Figure 3C) is a marker for nematodes with nuclear localization of this transcription factor (requirement for activation). Treatment of the nematodes with different concentrations of eAE for a short incubation time (1 h) resulted in no significant change in the intracellular localization of DAF-16 (Figure 3A). 

### 3.4. Requirement of DAF-16 for eAE-Mediated Effects

**Figure 4:**
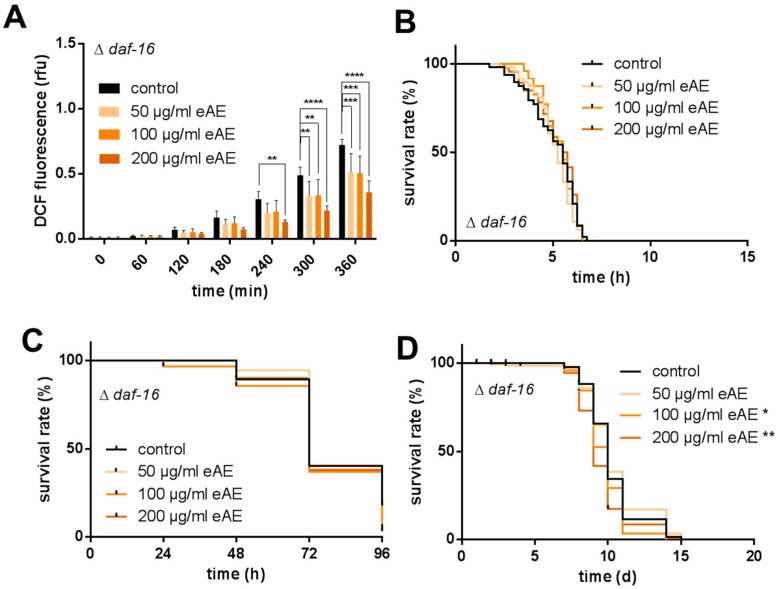
Requirement of DAF-16 for the protective effects of eAE. (**A**) Thermally induced increase in ROS (DCF assay): L4 larvae deficient in DAF-16 were treated with different concentrations of eAE or with DMSO (vehicle, 0.4%) for 24 h at 20 °C. After washing, they were transferred individually into the wells of a 384-well plate containing 50 µM H_2_DCF-DA. The increase in fluorescence was measured at 37 °C. Values are mean ± SD, n = 3 (each 16 individuals per group), two-way ANOVA with Dunnett’s multiple comparisons test vs. control at the correspondent time point, ** *p* ≤ 0.01, *** *p* ≤ 0.001, **** *p* ≤ 0.0001; (**B**) Thermal stress resistance: L4 larvae deficient in DAF-16 were treated with different concentrations of eAE or with DMSO (vehicle, 0.4%) for 24 h at 20 °C. After washing, they were transferred individually into the wells of a 384-well plate containing 1 µM SYTOX^®^ Green. The increase in fluorescence was measured at 37 °C. The graph shows the percentage of viable nematodes. Kaplan-Meier statistics were used for the comparison of the survival curves, n = 3 (each 16 individuals per group), Log Rank (Mantel-Cox) test; (**C**) Oxidative stress resistance: L4 larvae deficient in DAF-16 were incubated with eAE or with DMSO (vehicle, 0.4%) for 72 h at 20 °C. Then, all nematodes were transferred into extract-fee media containing 50 mM paraquat. Every 24 h, the survival of the nematodes was surveyed via touch-provoked movement. Kaplan-Meier statistics were used for the comparison of the survival curves, n = 3 (each 40 individuals per group), Log Rank (Mantel-Cox) test; (**D**) Modulation of life span: L4 larvae deficient in DAF-16 were incubated with different concentrations of eAE or DMSO (vehicle, 0.4%). On five days a week, media were exchanged and survival of the nematodes was tested via touch-provoked movement. Kaplan-Meier statistics were used for the comparison of the survival curves, n = 3 (each 40 individuals per group), Log Rank (Mantel-Cox) test, * *p* ≤ 0.05, ** *p* ≤ 0.01. Calculated data for thermal and oxidative stress resistance, as well as lifespan, are shown in Supplementary Tables S1–S3.

Even if the eAE showed no direct effect on the localization of the transcription factors DAF-16 after a short-time incubation (1 h), this signaling pathway may be necessary to mediate the antioxidative and stress-protecting effects of the extract shown after long-time incubation (24 h). To analyze this hypothesis, we used a *C. elegans* strain with a defect in DAF-16 (CF1038: DAF-16 loss of function). Using this strain, eAE was still able to reduce the thermal induced ROS generation significantly, suggesting that this transcription factor is not necessary to mediate this effect (Figure 4A). However, the protective effect of eAE against thermal-induced death of the nematodes is completely abolished in the loss of function strain, demonstrating the necessity of DAF-16 (Figure 4B). The same was detectable for the protection against paraquat-induced stress and the life span-prolonging effect (Figure 4C,D).

## 4. Discussion

It is well-known that extracts from *Agrimonia* spp. possess a high radical scavenging capacity, which is measured via the reduction of colored radicals (ABTS: 2,2-azinobis (3-ethylbenzothiazoline-6-sulfonic acid); DPPH: 2,2-Diphenyl-1-picrylhydrazyl) *in vitro* [3,9]. It is also known that extracts from *Agrimonia* spp. are able to protect against oxidative stress *in vivo* [9]. The aim of our study was to investigate the antioxidative effects of an agrimony extract in greater detail to show, e.g., dose-dependent effects *in vivo*, as well as molecular mechanisms of the effects using the model organism *Caenorhabditis elegans*. Antioxidative effects of plant compounds/plant extracts *in vivo* may be directly mediated via radical scavenging effects mediated by phenolic groups (as detectable in the TEAC assay). On the other hand, plant compounds/plant extracts may also cause indirect antioxidative effects via an interference with intracellular signaling processes [10,11]. These indirect effects will result in an increased expression of antioxidative enzymes like superoxide dismutase or catalase. A pivotal transcription factor for the expression of antioxidative enzymes is the DAF-16 transcription factor, a downstream component of the insulin-like signaling pathway [12,13]. As an orthologue of mammalian FoxO proteins, DAF-16 is inactivated after insulin signaling via a shuttle from the nucleus to the cytosol. This key transcription factor integrates different signals from the insulin/IGF-1 signaling pathway, mammalian target of rapamycin (mTOR) signaling, AMP-activated protein kinase (AMPK) pathway, c-Jun N-terminal kinase (JNK) pathway, and germline signaling to modulate aging and longevity [13].

In our model system, we were able to show both a protection against oxidative stress (paraquat) and a decrease in ROS formation *in vivo* (DCF assay). However, this decrease in ROS formation was not mediated by a modulation of DAF-16 since antioxidative effects were still detectable in DAF-16 loss-of-function animals. The reduction of ROS caused by eAE may be mediated by the strong radical scavenging effect of the constituents of the extract. Other authors reported an interference of agrimony compounds with specific components of various cellular signal transduction pathways. For example, Wang et al. showed that agrimol B, a polyphenol derived from *Agrimonia pilosa* Ledeb., significantly induced cytoplasm-to-nucleus shuttle of SIRT1, a histone deacetylase [14]. Since this enzyme is involved in regulating the life span of organisms, we further analyzed the effect of eAE on the life span of *C. elegans*. In our study, we were the first to show that this plant is able to promote longevity: the high concentrations tested (100 and 200 µg/mL) increased the life span of the nematodes. Additionally, we were able to show that this effect is strongly dependent on the transcription factor DAF-16, since in nematodes without this factor (loss-of-function animals), no prolongation of life span was detectable. Even more, eAE resulted in a decrease in life span of these loss-of-function animals. We conclude that this transcription factor is important in mediating the effects of eAE. Since it is known that DAF-16 has pleiotropic functions in the nematode, we analyzed whether it is necessary for the other effects reported in our study. This was the case: both the protection against thermal stress and oxidative stress was completely dependent on DAF-16. Since this factor is also a component of the insulin-like signaling pathway, eAE may be interesting for the treatment of diabetes and related metabolic diseases.

This connection is not new: A first correlation between agrimony and diabetes was reported in 1990 by Swanston-Flatt et al. [15]. They showed that agrimony is able to reduce the level of hyperglycaemia during the development of streptozotocin diabetes in mice, at least mediated via antioxidant effects [15]. The involvement of distinct bioactive compounds from *Agrimonia* spp. and insulin resistance was shown more recently by Teng et al. They demonstrated that agrimonolide and desmethylagrimonolide isolated from *Agrimonia pilosa* Ledeb. were able to modulate the glucose metabolism in insulin-resistant HepG2 cells: both compounds elevated glucokinase activity, reduced glucose-6-phosphatase activity, and increased the insulin-mediated glycogen level in hepatocytes [16]. Wang et al. demonstrated that agrimol inhibited the differentiation of the adipocytes, as well as adipogenesis [14]. Beneficial effects of an aqueous *Agrimonia* extract were also reported in an animal model showing that this extract improves the impaired glucose tolerance in high-fat diet-fed rats by decreasing the inflammatory response [17]. 

In our experimental model system, we were able to show that the occurrence of a transcription factor downstream of the insulin signaling pathway is essential for mediating the beneficial effects of eAE in the nematode. However, we were not able to show the interference between eAE and the DAF-16 transcription factor directly (no significant change in intracellular localization after incubation with eAE), but maybe the incubation time (1 h) was too short. 

Another point which was not observed in our experimental system was protection against amyloid-β-toxicity. Since *Agrimonia* spp. are rich in secondary plant compounds, it has been suggested that this plant should protect against neurodegenerative diseases like Alzheimer’s disease. It was shown that distinct phytochemicals isolated from *Agrimonia pilosa* Ledeb. were able to inhibit the acetylcholine esterase [18,19]. Kubinova et al. showed that an extract of *A. procera* (100 μg/mL) also displayed the inhibition of acetylcholine esterase [20]. Lee et al. reported that an extract of *Agrimonia eupatoria* attenuated glutamate-induced oxidative stress in hippocampal cells [21]. Park et al. demonstrated that water extracts of *Agrimonia pilosa* Ledeb. prevented amyloid-β (25-35)-induced cell death in PC12 cells. Furthermore, the extract improved cognitive dysfunction and glucose homeostasis in rats with experimentally-induced AD-type dementia (hippocampal infusions of plaque-forming amyloid-β (25-35) or non-plaque forming amyloid-β (35-25)) [22]. We used *Caenorhabditis elegans* as a simple model to evaluate the protective effects of eAE against amyloid-β-toxicity. However, in this model, no protection against amyloid-β-toxicity was detectable (up to 200 µg/mL), while caffeine as a positive control strongly protected the animals from the toxic amyloid stress. The discrepancy between the nematode and rat may be due to the simplified model, since only a specific amyloid-β-form is expressed. However, caffeine as a positive control resulted in strong protection; furthermore, acetylcholine esterase inhibitors are able to cause protection in our experimental system (aldicarb, donepezil; data not shown). Maybe the amount of phytochemicals with acetylcholinesterase-inhibiting properties was too low in the extract concentration used (200 µg/mL).

## 5. Conclusions

*Agrimonia procera* is a pharmacologically used plant rich in antioxidative phytochemicals, but studies concerning the mechanistical effects of this plant are limited. Besides the strong radical-scavenging activity of an *Agrimonia procera* extract, we were able to demonstrate antioxidative effects, as well as a dose-dependent protection against oxidative and thermal stress and a prolongation of life span, in the model organism *Caenorhabditis elegans*. Furthermore, we demonstrated that these protective effects were, at least in part, dependent on the transcription factor DAF-16 (FoxO orthologue). Further experiments are needed to analyze the effect of the extract on the expression of antioxidative enzymes (e.g., superoxide dismutase, catalase). We conclude that the *Agrimonia procera* extract not only causes radical scavenging effects, but also interferes with distinct components of the signal transduction. This interaction with a component of the insulin-like signaling pathway makes the plant interesting for the treatment of metabolic diseases and diabetes.

## Supplementary Materials

The following are available online at http://www.mdpi.com/2076-3921/7/12/192/s1, Supplementary table S1. Modulation of life span induced by different concentration of eAE vs. control (DMSO), Supplementary table S2. Modulation of thermal stress resistance induced by different concentration of eAE vs. control (DMSO), Supplementary table S3. Resistance against paraquat-induced oxidative stress: Modulation by different concentration of eAE vs. control (DMSO), Supplementary table S4. Resistance against amyloid-beta-induced stress: Modulation by different concentration of eAE vs. control (DMSO).
