# Supplementary material for: Agrimonia procera Wallr. Extract Increases Stress Resistance and Prolongs Life Span in Caenorhabditis elegans via Transcription Factor DAF-16 (FoxO Orthologue)
Source: Antioxidants (Basel). 2018 Dec 14;7(12):192. doi: 10.3390/antiox7120192 (PMC6315603; doi:10.3390/antiox7120192)
Supplement: Supplementary file 1 [file antioxidants-07-00192-s001.pdf]

Supplementary table S1. Modulation of life span induced by different concentration of eAE vs. control (DMSO).

Genotype	Treatment	Mean [d] ± SD	Difference to Control %	Median [d] ± SD	Difference to Control %	p-value vs. Control
wild type	DMSO	17.3 ± 0.430		17.0 ± 0.267		
	eAE 50 µg/mL	18.7 ± 0.409	+ 8.1	18.0 ± 0.533	+ 5.9	0.002
	eAE 100 µg/mL	19.5 ± 0.322	+ 12.7	21.0 ± 0.519	+ 23.5	0.0001
	eAE 200 µg/mL	18.6 ± 0.414	+ 7.5	18.0 ± 0.734	+ 5.9	0.014
CF1038 (mu86) [Δ*daf-16*]	DMSO	10.2 ± 0.164		10.0 ± 0.142		
	eAE 50 µg/mL	10.3 ± 0.209	+ 1.0	10.0 ± 0.177	± 0	0.474
	eAE 100 µg/mL	9.7 ± 0.130	- 4.9	10.0 ± 0.156	± 0	0.035
	eAE 200 µg/mL	9.5 ± 0.171	− 6.9	9.0 ± 0.151	− 10.0	0.001

Supplementary table S2. Modulation of thermal stress resistance induced by different concentration of eAE vs. control (DMSO).

Genotype	Treatment	Mean [h] ± SD	Difference to Control %	Median [h] ± SD	Difference to Control %	p-value vs. Control
wild type	DMSO	5.0 ± 0.196		5.3 ± 0.433		
	eAE 50 µg/mL	4.8 ± 0.258	− 4.0	5.5 ± 0.522	+ 3.8	0.873
	eAE 100 µg/mL	5.7 ± 0.210	+ 14.0	6.0 ± 0.071	+ 13.2	0.002
	eAE 200 µg/mL	6.1 ± 0.166	+ 22.0	6.3 ± 0.108	+ 18.9	0.0004
	DMSO	5.0 ± 0.181		5.5 ± 0.286		
CF1038 (mu86)	eAE 50 µg/mL	5.1 ± 0.142	+ 2.0	5.3 ± 0.215	− 3.6	0.413
[Δ*daf-16*]	eAE 100 µg/mL	5.3 ± 0.124	+ 6.0	5.5 ± 0.133	± 0	0.857
	eAE 200 µg/mL	5.0 ± 0.221	± 0	5.5 ± 0.324	± 0	0.719

Supplementary table S3. Resistance against paraquat-induced oxidative stress: Modulation by different concentration of eAE vs. control (DMSO).

Genotype	Treatment	Mean [h] ± SD	Difference to Control %	Median [h] ± SD	Difference to Control %	p-value vs. Control
wild type	DMSO	70.2 ± 1.684		72.0 ± 1.858		
	eAE 50 µg/mL	75.6 ± 1.573	+ 7.7	72.0 ± 2.101	± 0	0.040
	eAE 100 µg/mL	76.9 ± 1.617	+ 9.5	72.0 ± 2.126	± 0	0.007
	eAE 200 µg/mL	77.7 ± 1.545	+ 10.7	72.0 ± 2.221	± 0	0.002
CF1038 (mu86) [Δ*daf-16*]	DMSO	79.1 ± 1.446		72.0 ± 2.228		
	eAE 50 µg/mL	80.4 ± 1.299	+ 1.6	72.0 ± 2.031	± 0	0.802
	eAE 100 µg/mL	76.6 ± 1.683	− 3.2	72.0 ± 2.193	± 0	0.028
	eAE 200 µg/mL	78.0 ± 1.596	− 1.4	72.0 ± 2.074	± 0	0.041

Supplementary table S4. Resistance against amyloid-beta-induced stress: Modulation by different concentration of eAE vs. control (DMSO).

Genotype	Treatment	Mean [h] ± SD	Difference to Control %	Median [h] ± SD	Difference to Control %	p-value vs. Control
CL4176 (cc546)	DMSO	30.8 ± 0.186		30.0 ± 0.276		
	eAE 50 µg/mL	30.9 ± 0.165	+ 0.3	30.0 ± 0.248	± 0	0.608
	eAE 100 µg/mL	31.0 ± 0.165	+ 0.6	30.0 ± 0.253	± 0	0.587
	eAE 200 µg/mL	30.9 ± 0.172	+ 0.3	30.0 ± 0.222	± 0	0.519
	Caffeine 5 mM	33.7 ± 0.075	+ 9.4			< 0.0001
